# Cohort Profile Update: The 1970 British Cohort Study (BCS70)

**DOI:** 10.1093/ije/dyac148

**Published:** 2022-07-18

**Authors:** Alice Sullivan, Matt Brown, Mark Hamer, George B Ploubidis

**Affiliations:** Centre for Longitudinal Studies, Social Research Institute, University College London, London, UK; Centre for Longitudinal Studies, Social Research Institute, University College London, London, UK; Division of Surgery and Interventional Science, Faculty of Medical Sciences, University College London, London, UK; Centre for Longitudinal Studies, Social Research Institute, University College London, London, UK

Key Features

- The 1970 British Cohort Study (BCS70) is an ongoing, multidisciplinary, longitudinal study of a cohort of over 17 000 births in England, Scotland and Wales.
- The initial sample comprised all births in Britain in a single week in 1970.
- Fifty years of follow-up provide opportunities for new research on social, economic and health outcomes in mid-life, their antecedents and generational change.
- In the most recent face-to-face survey at age 46, 8581 study members took part. This included a survey interview, a range of bio-measures administered by a nurse, an online dietary diary and physical activity and sedentary behaviour monitoring using thigh-worn accelerometry.
- Three COVID-19 web surveys were carried out over 2020–21.
- BCS70 datasets are accessible via the UK Data Service: further information can be found at [https://cls.ucl.ac.uk/cls-studies/1970-british-cohort-study/].

## The original cohort

The 1970 British Cohort Study (BCS70) began in 1970 with data collection on the births and social circumstances of over 17 000 babies born in the UK. Cohort members who were born in Northern Ireland were included in the birth survey but dropped from the study in all subsequent sweeps. At the time of writing, the cohort members are in their early fifties.

The initial BCS70 birth study had a particular focus on perinatal mortality.^1^^,^^2^ The focus of the study has broadened over time, reflecting the interest of both social science and health disciplines in each life stage, as the cohort has moved through childhood into adolescence, adulthood and mid-life. Sullivan, Brown and Bann describe the history and context of the cohort from birth to mid-life.^3^

BCS70 is the third of a series of UK national birth cohorts which began with the 1946 National Survey of Health and Development (NSHD),^4^ followed by a second cohort in 1958, the National Child Development Study (NCDS).^5^ The Millennium Cohort Study^6^ began 30 years after BCS70. The Centre for Longitudinal Studies at University College London houses the 1958, 1970 and 2000 cohorts.

Elliott and Shepherd^7^ describe the BCS70 study up to 2004, when the cohort members were aged 34. BCS70 began as the British Births Survey, under the directorship of Roma and Geoffrey Chamberlain. Data collection at birth (1970) was conducted by midwives. Subsequent surveys at the ages of 5 (1975) and 10 years (1980) took place under the auspices of the Department of Child Health at the University of Bristol, led by Neville Butler. Butler set up the International Centre for Child Studies (ICCS) which carried out the age-16 (1986) survey. Following a 10-year hiatus, the next major wave of data collection was at age 26 (1996), led by John Bynner at the Social Statistics Research Unit (SSRU), which subsequently became the Centre for Longitudinal Studies now based at University College London. This was followed by a face-to-face survey at age 30 (2000) carried out simultaneously with data collection at age 42 for the 1958 cohort (NCDS), with 90% of questions shared between the two cohorts. Heather Joshi directed the age-34 (2004) survey, a face-to-face interview including adult literacy and numeracy assessments. Jane Elliott become Principal Investigator of the study in 2005, followed by Alice Sullivan in 2010 and George Ploubidis in 2021. Mark Hamer was joint Principal Investigator with Alice Sullivan of the age-46 biomedical study.

## What is the reason for the new data collection?

Subsequent surveys have been carried out at regular 4-yearly intervals in order to track the lives of the study members into mid-life, with the intention of continuing to follow them into old age. This is designed to provide the opportunity to understand the precursors of beneficial and adverse circumstances in mid-life and their consequences in subsequent older age, maximizing the value of the earlier data collections. A summary of new data collection is shown in Table 1. Planned surveys of the whole cohort have been completed at ages 38, 42 and 46.

**Table 1:** Summary of new data collection

Survey	Year	Data collected from	Achieved sample	Funder
BCS70 Age 38 Survey	2008	Cohort members	8874	ESRC
BCS70 Age 42 Survey	2012	Cohort members	9841	ESRC
BCS70 Age 46 Survey	2016–18	Cohort members	8581	ESRC, MRC, BHF
COVID-19 Web Survey Wave 1	2020 (May)	Cohort members	4223	ESRC
COVID-19 Web Survey Wave 2	2020 (October)	Cohort members	5320	ESRC
COVID-19 Web Survey Wave 3—with telephone follow-up	2021 (February)	Cohort members	5758	ESRC
COVID-19 Antibody Testing	2021 (March)	Cohort members	2547	MRC

ESRC, Economic and Social Research Council; MRC, Medical Research Council; BHF, British Heart Foundation.

The Age 38 Survey consisted of a 25-min telephone interview and sought to establish changes in circumstances since the previous interview. The topics covered included: household situation, housing, relationships, children and wider family, family income and wealth, employment, lifelong learning, health and health behaviour.

The Age 42 Sweep involved a 60-min interview and a paper self-completion questionnaire. It aimed to provide rich data on the cohort members’ lives across a wide range of domains. Topics covered included relationships, children, parents, place of residence, economic activity, income, qualifications and training, physical and mental health, smoking, drinking, diet, exercise, identity, attitudes and values, religion and leisure activities. A vocabulary test was also administered.

The Age 46 Survey included a full range of bio-measures, including for the first time collection of blood samples administered by a nurse. The inclusion of objective measures of health was designed to allow researchers to assess the longitudinal predictors of health in mid-life. Many of the measures were designed to allow for cross-cohort comparisons with the bio-measures administered to NCDS in mid-life.

The Age 50 Survey, due to commence in 2020, was delayed by the pandemic and started in September 2021. The pandemic has prompted new data collection in order to understand its impact on study members’ lives. Three surveys collecting information about the impact of the pandemic were conducted in May 2020, October 2020 and February 2021. The COVID-19 Surveys were also completed by participants in NSHD, NCDS, the Millennium Cohort Study and Next Steps—the first simultaneous data collection across these UK cohorts. In March 2021, participants provided blood samples which were tested for COVID-19 antibodies.

## What will be the new areas of research?

A number of new areas of research have been generated by the new data:

- Investigation of the antecedents of mid-life circumstances, including the long-range influence of early life circumstances on mid-life social and economic circumstances and physical and mental health;
- cross-cohort comparisons of mid-life between the 1946, 1958 and 1970 cohorts;
- investigation of the determinants of objectively measured biomarkers in mid-life, including sedentary behaviour;
- investigation of experiences of the COVID-19 pandemic, exploiting rich prior data.

## Who is in the cohort?

Participants are survivors from the original sample of over 17 000 births, all born in the UK during 1 week in 1970. During childhood, cohort members were traced through schools, and immigrants born in the reference week were added to the target sample. Efforts have been made to maintain participant engagement through feedback mailings, birthday cards, study websites and social media. Efforts are made to trace lost participants through use of study records, internet searches, electoral records and administrative databases, but failure to trace individuals when they move is the main cause of attrition over time.

At the most recent major wave of data collection, in 2016, the cohort members were aged 46. Due to selective attrition, the sample includes more cohort members from an advantaged childhood socioeconomic background (27.1% non-manual paternal social class at birth, 32.2% at age 46) and more women (48.2% at birth, 52.0% at age 46). Nevertheless, recent work from the Centre for Longitudinal Studies has shown that capitalizing on observed variables from earlier waves allows researchers to replicate the original distribution of the baseline sample, reduce bias and restore sample representativeness.^8^^,^^9^

## What has been measured?

Measurements collected in the three main waves of data collection from age 38 onwards are listed in Table 2, and the COVID-19 survey content in Table 3.

**Table 2:** Summary of content—Age 38, Age 42 and Age 46 Surveys

	Age 38 Survey (telephone): 2008	Age 42 Survey: 2012	Age 46 Survey: 2016
**Family**			
Household composition	*	*	*
Cohabiting relationships	*	*	*
Non-cohabiting relationships	*	*	*
Relationship satisfaction		*	*
Pregnancies and births	*	*	*
Children	*	*	*
Absent children—where living, contact, maintenance payments		*	
Older children—economic activity status, education, marital status, fertility		*	
Fertility intentions and childlessness		*	
Use of fertility treatments		*	
Parents (including provision of care)	*	*	*
Grandchildren			*
Social contact and social support		*	*
Sharing of domestic chores		*	
**Housing**			
Housing history	*	*	*
Current home (accommodation type, number of rooms, tenure)	*	*	*
Housing wealth		*	
Housing expenses		*	
Satisfaction with home		*	
**Employment/economic activity**			
Economic activity history	*	*	*
Current economic activity status	*	*	*
Current occupation	*	*	*
Hours	*	*	*
Job satisfaction		*	*
Job security		*	*
Travel to work		*	*
Work-life balance		*	*
Job demands			*
Help to find work from parents, relatives and friends		*	
Partner economic activity status	*	*	*
Partner occupation		*	
**Financial**			
Financial situation	*	*	*
Earnings from employment	*	*	*
Partner earnings from employment		*	
Income from benefits		*	
Income from other sources		*	
Total household income	*	*	*
Pensions		*	
Savings		*	*
Debt		*	*
Car ownership		*	*
**Education**			
Qualifications	*	*	*
Training		*	
**Cognition**			
Vocabulary assessment		*	
Memory, executive function, attention			*
**Physical health**			
General health (single question)	*	*	*
General health (SF-36)			*
Health conditions	*	*	*
Long-standing illness		*	*
Dental health			*
Medication			*
Menopause		*	*
Height (self-reported)		*	*
Weight (self-reported)		*	*
Height (measured)			*
Weight (measured)			*
Body fat			*
Hip and waist circumference			*
Maximal grip strength			*
Leg-raise/balance			*
Blood sample collection (analysis of cholesterol, HbA1c, triglycerides and C-reactive protein, DNA to be extracted in 2022)			*
**Mental health and well-being**			
Warwick-Edinburgh Mental Wellbeing Scale		*	*
Malaise scale		*	*
Life satisfaction		*	*
Self efficacy		*	
**Lifestyle**			
Smoking	*	*	*
Alcohol consumption (past 7 days)		*	*
Problematic alcohol consumption—AUDIT scale		*	*
Exercise/physical activity (self-reported)		*	*
Physical activity (measured with accelerometer)			*
Sleep		*	*
Diet		*	*
Screen-time/computer use		*	*
Reading			*
Leisure activities		*	
**Identity, attitudes, social and political participation**			
Sexuality		*	
Class identity		*	
Voting		*	*
Trust		*	
Political participation		*	
Religion			
Membership of organizations		*	*

AUDIT, Alcohol Use Disorders Identification Test.

**Table 3:** Summary of content: COVID-19 surveys—Waves 1 to 3

	Wave 1 (May 2020)	Wave 2 (September 2020)	Wave 3 (February 2021)
**COVID-19, vaccination and health care**			
Exposure to COVID-19	*	*	*
Long COVID-19 symptoms			*
Symptoms of COVID-19	*	*	*
Compliance with social distancing guidelines	*		*
Whether has downloaded NHS Track & Trace app			*
Vaccination			*
Self-rated general health	*	*	*
Long-standing health conditions	*	*	*
Disruption to health care	*	*	*
**Time use**			
Time use on typical weekday since outbreak	*	*	
**Family and household**			
Household composition	*	*	*
Changes in household composition	*	*	*
Children	*	*	*
Changes in childcare and schooling arrangements	*	*	*
Whether in non-cohabiting relationship	*	*	*
Relationship satisfaction and family conflict	*	*	*
Household care needs and receipt of care	*	*	*
**Housing**			
Number of rooms	*	*	*
Access to garden	*	*	*
Tenure	*	*	*
**Financial situation**			
Financial situation	*	*	*
Food security, use of food banks	*		
Benefit receipt	*	*	*
Use of mortgage/rent/debt holidays since outbreak	*	*	*
Giving/receiving financial help		*	*
Total household income (pre-pandemic and current)			*
Employment and education			
Economic activity, pre-COVID and current (cohort member and partner)	*	*	*
**Employment, pre-COVID (cohort member and partner)**			
Occupation, hours, contract type	*	*	*
Gross pay			*
**Employment, current (cohort member and partner)**			
Occupation, hours, contract type, work location	*	*	*
Gross pay			*
Key worker status	*		*
Job satisfaction		*	*
Home-working satisfaction		*	*
**Education (cohort member only)**			
Education during pandemic	*	*	*
Disruption of teaching/online learning	*	*	*
Satisfaction with learning provision/academic progress	*	*	*
**Health behaviours**			
Smoking and vaping	*	*	*
Alcohol consumption	*	*	*
Physical activity	*	*	*
Diet (fruit and veg)	*	*	*
Sleep	*	*	*
Weight	*	*	*
Screen time			*
**Social contact, social support and loneliness**			
Contact with friends and family	*	*	*
Participation in community activity	*	*	*
Provision of help to others	*	*	*
Social support	*	*	*
Loneliness	*	*	*
**Mental health**			
Life-satisfaction	*	*	*
Self-assessed mental health		*	*
Control over life		*	*
Mental health (Malaise scale, GAD-2, PHQ-2)	*	*	*
**Optimism, risk, patience and trust**			
Optimism		*	*
Risk, patience and trust	*	*	*
Trust in government and political leaders	*	*	*
**Life events**			
Adverse life events		*	*
**Consent to link to ZOE tracker app**	*	*	*

GAD_2, Generalised Anxiety Disorder (2 item); PHQ-2, Patient Health Questionnaire (2 item).

The Age 38 Survey contained core measures only, due to time constraints.

Novel measures at age 42 included: sexuality (asked for the first time at this wave), reading behaviour and a vocabulary assessment (both included for the first time since age 16). Cohort members were asked for consent to link survey data with health and economic records held by the National Health Service, Department for Work and Pensions and Her Majesty’s Revenue and Customs. Hospital Episode Statistics have been linked, and are available via the UK Data Archive.

Novel measures at age 46 included: a range of bio-measures, cognitive assessments, thigh-worn accelerometry and a dietary diary. BCS70 is being genotyped at the time of writing.

The COVID-19 survey included assays for COVID-19 antibodies and consent for data linkage to the ZOE app.^10^

## What has it found? Key findings and publications

BCS70 represents a major resource for research in the social and health sciences. To date over 1000 publications have used BCS70 data and over 830 of these have been published since the publication of the 2006 cohort profile, as documented in the CLS Bibliography [https://www.bibliography.cls.ucl.ac.uk/]. Space permits us only to highlight a fraction of this literature. We outline some major strands of recent research below.

### Overweight and obesity

Cross-cohort analysis has demonstrated that during childhood the 1970 generation were no more likely to be overweight than generations born in 1946 and 1958, but became overweight from their 1980s adolescence onwards. Later-born generations have higher body mass index, with a substantial increase in childhood overweight and obesity compared with the 1970 generation.^11^ Exposure to obesity across adult life is related to cardiometabolic risk markers.^12^

### Mental health

Mid-life is a period of relatively high psychological distress, and individuals born in 1970 were more likely to experience psychological distress than those born in 1946 and 1958.^13^^,^^14^ Psychological distress in adulthood accompanies a doubled risk of premature mortality.^15^

### Physical activity

BCS70 self-reports of physical activity are complemented by a measure of sedentary time using a thigh-worn accelerometer at age 46.^16^ Device-assessed physical activity and sitting-time were associated with physical and mental health in mid-life.^17^^,^^18^

### Biomarkers

Blood collected at age 46 was used to measure a number of risk-markers. Secular trends in cholesterol have been observed,^19^ and prevalence of multimorbidity at age 46 was 33.8%.^20^

### Education, learning, social mobility and work

Advantaged social origins and schooling have life course advantages in terms of educational and occupational attainment.^21^^,^^22^ Reading for pleasure is linked to higher cognitive scores in adolescence and adulthood, controlling for earlier scores.^23^^,^^24^ The pay gap between men and women has reduced for BCS70 compared with earlier generations.^25^

## What are the main strengths and weaknesses?

The strengths of the study include its large, nationally representative sample. It provides prospective, longitudinal data, with follow-up spanning a large portion of the life course. It contains extensive and broad data coverage which allows multidisciplinary research linking data across life domains, such as economic, social and health factors. Finally, BCS70’s comparability with earlier and later UK cohorts allows cross-cohort comparisons.

Weaknesses include selective attrition, which is a consideration for all longitudinal studies. In addition, gaps in the series of national cohorts limit the scope for comparison across generations, as some generations are missing from the series. BCS70 was the third in a series of UK birth cohorts spaced 12 years apart (1946, 1958 and 1970). The intention was to continue the series at 12-year intervals, but a lack of political will and funding meant that 30 years passed before the next cohort emerged (the Millennium Cohort Study). The cancellation of a planned 2012 cohort led to a further gap in the series. However, the Early Life Cohort (ELC) and Children of the 2020s (COTS2020) are incipient additions to the birth cohorts series.

## Can I get hold of the data? Where can I find out more?

The Centre for Longitudinal Studies (CLS) is responsible for overseeing all aspects of the management of BCS70. CLS is an Economics and Social Research Council resource centre and offers support and advice to data users. The CLS website provides detailed documentation and information on research projects and publications arising from the study [https://cls.ucl.ac.uk/cls-studies/1970-british-cohort-study/]. Data from the 1970 cohort are held and distributed by the UK Data Service, and are available to bona fide researchers who sign an undertaking regarding proper use of the data [https://beta.ukdataservice.ac.uk/datacatalogue/series/series?id=200001]. M.B. is a senior survey manager and can be contacted for further information at [matt.brown@ucl.ac.uk].

## Ethics approval

Ethics approval is obtained from a National Health Service Research Ethics Committee in advance of each sweep of data collection. The Age 38 Survey was approved by Southampton & South West Hampshire Research Ethics Committee (08/H0504/144), the Age 42 Survey by London-Central Research Ethics Committee (11/LO/1560) and the Age 46 Survey by South East Coast—Brighton & Sussex (15/LO/1446). In addition, London-Central Research Ethics Committee have provided ethics approval for the ongoing activities of the study in between sweeps of data collection: keeping in touch and tracing study members; cleaning, documenting and providing access to the data for research; and linking data from administrative sources to survey data to increase the utility of the data for research (14/LO/0371).

## Author contributions

All authors contributed to writing the paper.

## Funding

We acknowledge funding from the Economic and Social Research Council, ES/M001660/1.
